# Supplementary material for: The nature of deep overturning and reconfigurations of the silicon cycle across the last deglaciation
Source: Nat Commun. 2020 Mar 24;11:1534. doi: 10.1038/s41467-020-15101-6 (PMC7093442; doi:10.1038/s41467-020-15101-6)
Supplement: Supplementary file 1 — Supplementary Information [file 41467_2020_15101_MOESM1_ESM.pdf]

Supplementary Information for:

The nature of deep overturning and reconfigurations of the silicon cycle across the last deglaciation

by Dumont et al.

Supplementary Note 1: Age models

The age models for each of the cores were constructed using an assortment of ^{14}C dating, $\delta^{18}O$ stratigraphic correlation and lithogenic material stratigraphic correlation. The tie-points applied to cores MD84-551, MD88-773 and MD88-772 are given in Supplementary Tables 1, 2 and 3, respectively. ^{14}C dating of picked planktic foraminifera (*Neogloboquadrina pachyderma*) was performed for each of the three cores by Accelerator Mass Spectrometry (AMS) at the NERC Radiocarbon Laboratory, East Kilbride. Modern reservoir ages estimated using the GLODAP database[1] are 1100 years ($\Delta^{14}C$ = -128 ‰), 990 years ($\Delta^{14}C$ = -116 ‰) and 840 years ($\Delta^{14}C$ = -100 ‰) for MD84-551, MD88-773 and MD88-772, respectively. ^{14}C dates were calibrated using the Calib 7.04 program[2] with the Marine13 calibration curve. The reservoir corrections were kept constant throughout the records.

**Depth (cm)**	**Tie-point age (ka)**	**Description and justification**	**^{14}C age (^{14}C ka BP) +/- 1σ**
75	9.655	^{14}C (ΔR = 700 years) SUERC-73145	9.698 +/- 0.037
120	11	*N. pachyderma* $\delta^{18}O$ (see Supplementary Figure 2)<br>First large steep increase in $\delta^{18}O$ before the earliest ^{14}C datum at 9.655 ka. Tied to the end of the deglacial decrease in $\delta^{18}O$ in MD12-3396CQ.	N/A
143	13.4	*N. pachyderma* $\delta^{18}O$ (see Supplementary Figure 2)<br>Centre of the rapid increase in $\delta^{18}O$ in MD12-3396CQ approximately half-way through the glacial-interglacial $\delta^{18}O$ increase.	N/A
155	14.5	*N. pachyderma* $\delta^{18}O$ (see Supplementary Figure 2)<br>Centre of the rapid decrease in $\delta^{18}O$ in MD12-3396CQ shortly before the rapid increase given in the tie-point above and after the ^{14}C datum at 16.624 ka	N/A
182	16.624	^{14}C (ΔR = 700 years)[4]	14.86 +/- 0.110
274	37.65	Magnetic susceptibility minimum prior the broad peak during the last glacial maximum (see Supplementary Figure 3).	N/A

Supplementary Table 1. Age control points applied to core MD84-551.

**Depth (cm)**	**Tie-point age (ka)**	**Description and justification**	**^{14}C age (ka BP) +/- 1σ**
120	3.748	^{14}C (ΔR = 590 years) SUERC-73133	4.392 +/- 0.036
170	5.922	^{14}C (ΔR = 590 years) SUERC-73134	6.126 +/- 0.038
215	6.499	^{14}C (ΔR = 590 years) SUERC-73135	6.666 +/- 0.037
255	8.787	^{14}C (ΔR = 590 years) SUERC-73136	8.841 +/- 0.039
275	9.52	^{14}C (ΔR = 590 years) SUERC-73140	9.466 +/- 0.038
295	9.975	^{14}C (ΔR = 590 years) SUERC-73141	9.762 +/- 0.038
335	12.992	^{14}C (ΔR = 590 years) SUERC-73143	12.103 +/- 0.041
353	14	Steepest point of the decrease in magnetic susceptibility half-way through the deglacial decline in MD12-3396CQ (see Supplementary Figure 3).	N/A
550	21.8	Steepest point of the short decrease in magnetic susceptibility at the mid-point of the broad glacial high in magnetic susceptibility in MD12-3396CQ. This is decline is more visible in MD94-103 and MD88-769 (see Supplementary Figure 3).	N/A
803	37.65	Magnetic susceptibility minimum prior the broad peak during the last glacial maximum (see Supplementary Figure 3).	N/A

Supplementary Table 2. Age control points applied to core MD88-773.

Depth (cm)	Tie-point age (ka)	Description and justification	^{14}C age (ka BP) +/- 1σ
18	7.209	^{14}C (ΔR = 400 years) SUERC-73123	7.075+/- 0.037
26	8.489	^{14}C (ΔR = 400 years) SUERC-73124	8.437+/- 0.036
37	10.588	^{14}C (ΔR = 400 years) SUERC-73125	10.088+/- 0.038
44	12.571	^{14}C (ΔR = 400 years) SUERC-73126	11.411+/- 0.039
48	13.159	^{14}C (ΔR = 400 years) SUERC-73131	12.062+/- 0.042
60	15.7	Steepest point of initial large deglacial decline in magnetic susceptibility prior to a slight pause in the decline and the ^{14}C datum on MD88-772 at 12.992 ka (see Supplementary Figure 3).	N/A
89	21.8	Brief minimum in magnetic susceptibility following the mid-glacial decline (see (2) above) and prior to the small increase during the late-glacial. Most visible in MD88-769 (see Supplementary Figure 3).	N/A
260	37.65	Magnetic susceptibility minimum prior the broad peak during the last glacial maximum (see Supplementary Figure 3).	N/A

Supplementary Table 3. Age control points applied to core MD88-772.

Carbonate preservation was poor within the early deglacial and LGM sections of MD88-773 and MD88-772, hence no ^{14}C dates are available for these periods. The age models of the remainder of these two cores were constrained using lithogenic detritus stratigraphy, correlating the lithogenic material contents of the two cores with magnetic stratigraphy records from nearby records with independent and ^{14}C-constrained age models. Correlating lithogenic material contents between records has been used previously in the Southern Ocean[8]. The flux of detrital material to the eastern Indian sector has been shown to be dominated by erosion of material from the Kerguelen Plateau[9]. Downcore variability in lithogenic material flux has been shown previously to be synchronous between numerous records across the Indian sector[6], suggesting a common regional control. The independently ^{14}C dated magnetic susceptibility records of MD12-3396CQ[5, 7], MD94-103[6] and MD88-769[6] are well-aligned, providing good evidence that changes in lithogenic material flux are regionally synchronous. Hence, the early-deglacial and LGM sections of MD88-773 and MD88-772 were graphically aligned to the three dated magnetic susceptibility records given above. The age control-points are shown in Supplementary Figure 3.

For MD84-551, the ^{14}C dates are supplemented with $\delta^{18}O$ and magnetic susceptibility stratigraphic correlation against MD12-3396CQ[5]. The tie-points applied are shown in Supplementary Figures 2 and 3.

Supplementary Note 2: Lithogenic fluxes and Fe fertilization in the Indian sector

The accumulation of lithogenic material in the sediments of the Indian sector of the Southern Ocean is primarily controlled by the erosion of bathymetric features such as the Kerguelen Plateau through both the glacial period and modern[9]. Hence, the variable silicification of diatom communities may be controlled at least in part by iron additions derived from the erosion of these features. Supplementary Figure 4 displays the Th-normalised fluxes of ^{232}Th (a commonly used proxy for lithogenic fluxes) and Fe in MD84-551 and MD88-773 along with the $\delta^{30}Si_{diat}$ data from the three cores. It is apparent that there is little correspondence between the ^{232}Th and Fe flux records and the $\delta^{30}Si_{diat}$ records, suggesting that much of the variability in the $\delta^{30}Si_{diat}$ through the deglaciation cannot be attributed to changes in lithogenic input, and thus changes in Fe fertilization, alone.

Supplementary Note 3: Sea ice and fronts

In the modern Southern Ocean, the uptake of DSi by diatoms and their deposition in sediments is focused to a circumpolar band known as the opal belt. Today the latitudinal limits of the opal belt are considered to be dictated by sea ice extent in the south and DSi concentrations in the north, which become limiting to diatom growth north of the polar front[12]. One could therefore argue that deglacial variability in the average sea ice extent and/or movement of the fronts may explain the $\delta^{30}Si_{diat}$ presented here.

It has been proposed that the front positions and average sea ice extent would have shifted poleward between the LGM and early Holocene. Although, topography would have restricted and steered the front movement in many locations of the Southern Ocean, including the Kerguelen region of Indian sector. A poleward shift of the fronts would have resulted in an increase in the $\delta^{30}Si_{diat}$ at any given impacted location. Therefore, the large decrease in $\delta^{30}Si_{diat}$ between the ACR and YD in the three cores cannot be explained by a poleward movement of fronts that one would expect during the Antarctic warming interval.

It should also be noted that the influence of DSi utilization by diatoms within sea ice has been shown to produce more extreme isotopic fractionations than observed by diatoms in open waters[13]. However, as suggested by Fripiat et al.[13], the contribution of sea ice diatoms to Southern Ocean sediments is minor and likely negligible with respect to the bulk isotopic composition. Furthermore, diatom-based reconstructions of sea ice presence[14] from MD88-773 and MD84-551 (Supplementary Figure 5) indicate that sea ice presence during the YD was comparable to that during the Holocene, yet the $\delta^{30}Si_{diat}$ within the three cores varied by as much as 0.9 ‰ between the two intervals. Hence, the $\delta^{30}Si_{diat}$ variability observed during the late deglaciation cannot be attributed to the influences of sea ice on silicon isotope fractionation by diatoms.

Supplementary Note 4: Diatom assemblage changes and $\delta^{30}Si_{diat}$

Culture experiments have suggested that some common Southern Ocean diatom species may have very contrasting Si isotope fractionation factors, ε (Sutton et al., 2013). Large changes in the relative abundance of particular species through a record could then lead to shift in the recorded $\delta^{30}Si_{diat}$ unattributable to changes in Si utilization or $\delta^{30}Si(OH)_4$. Sutton et al.[15] used the fractionation factors determined through culture experiments to predict the potential impact of species changes on the $\delta^{30}Si$ recorded in an Atlantic sector core (TN057-13-4PC)[16]. To do so, an estimation of the total bulk ε was made by scaling the empirically determined ε values of certain species by their relative abundance in a given sample. Sutton et al. focused on the variability of two taxa: the common open ocean taxon, *Fragiolopsis kerguelensis*, to which they assign an ε ($\varepsilon_{F.kerg}$) of -0.54 ‰, and *Chaetoceros brevis* to which they assign an ε ($\varepsilon_{C.brevis}$) of -2.09 ‰. The species effect of all other species combined was given an ε (ε_{other}) of -1.1 ‰. A cumulated fractionation factor (ε_{total}) can be reconstructed for each of the records based on the relative abundances (*A*) of the taxa.

$$\epsilon_{total} = \epsilon_{F.kerg}A_{F.kerg} + \epsilon_{C.brevis}A_{C.brevis} + \epsilon_{other}A_{other}$$

Where $A_{F.kerg}$ and $A_{C.brevis}$ are the fractional abundances of *F. kerguelensis and C. brevis*, respectively, and A_{other} is the fractional abundance of the remaining species.

The impact of changes in the cumulated fractionation factor were then interpreted by producing a reconstruction of the $\delta^{30}Si$ variability ($\delta^{30}Si_{model}$) based the equation of open system fractionation below.

$$\delta^{30}Si_{model} = \delta^{30}Si(OH)_4 + \epsilon_{total}$$

Where f is the fraction of DSi pool that remains unutilized. The value of f was set at 1, thus assuming instantaneous replenishment of the DSi pool (i.e. no utilization). In doing so the model will produce the most extreme ranges in $\delta^{30}Si_{model}$ attributable to changes in the bulk fractionation factor (ε_{total}) alone.

Supplementary Figures 6 and 7 show the $\delta^{30}Si_{model}$ results for MD84-551 and MD88-773, respectively, assuming an unchanging $\delta^{30}Si(OH)_4$ input of 1.7 ‰, along with their respective measured $\delta^{30}Si_{diat}$ data (emulating Figure 2 in [15]) and abundances of common diatom taxa in each record. The maximum possible species effect between the Holocene and Younger Dryas can be estimated using the difference between the maximum Holocene value given by the model (0.96 ‰ at 9.8 ka in MD84-551, 1.01 ‰ at 6.3 ka in MD88-773) and the minimum Younger Dryas value (0.87 ‰ at 12 ka in MD84-551, 0.96 ‰ at 12.6 ka in MD88-773). This produces a maximum species effect of 0.08 ‰ and 0.06 ‰ for MD84-551 and MD88-773, respectively. These values ae lower than the average external reproducibility of the $\delta^{30}Si_{diat}$ data (0.09 ‰) and as such we suggest that changes in ε made an insignificant contribution to the observed $\delta^{30}Si_{diat}$ excursion during the Younger Dryas.

The smaller species effect in the Indian sector records, compared to the TN057-13-4PC[15] can be attributed to the reduced variability in species composition. *F. kerguelensis* is the most abundant taxa throughout the deglacial sections of both MD88-773 and MD84-551. This contrasts to the record studied by Sutton et al.[15], where *F. kerguelensis* became the most abundant taxon only after the early deglaciation, before which the abundance of *C. brevis* approached that of *F. kerguelensis*. Based on the work by Sutton et al.[15] it appears that the high abundance of *C. brevis*, which they suggest has an ε that markedly deviates from the diatom average, may have biased the $\delta^{30}Si_{diat}$ record they presented. The Indian sector records presented here do not exhibit a large variability in *C. brevis* abundance that significantly biases the record. The $\delta^{30}Si_{diat}$ records are likely to be less sensitive to the variability of species other than *C. brevis* as their ε values have been shown to be much closer to the diatom mean[15].

Overall maximum ranges of the models are 0.28 ‰ and 0.25 ‰ for MD84-551 and MD88-773, respectively. Given that these estimates are the most extreme offsets possible attributed to purely changes in species abundance, we suggest that $\delta^{30}Si_{diat}$ biases related to species-dependent fractionation factors cannot explain the observed deglacial changes in $\delta^{30}Si_{diat}$.

Diatom assemblage data for MD88-772 is unavailable, however, given the proximity of this record to MD88-773 we do not expect any abrupt species shifts to occur within MD88-772 that can explain the $\delta^{30}Si_{diat}$ variability.

Supplementary Note 5: Mass balance model for quantifying DSi supply

$\delta^{30}Si_{diat}$ and opal fluxes can be applied to a mass balance model to demonstrate changes in DSi supply to the Southern Ocean surface and its leakage to lower latitudes. We follow the model created by Beucher et al.[17] which uses a single box each for the Antarctic and Subantarctic and estimates the silicon isotope, DSi and opal budgets of these boxes with constraints based on available data. Isotope fractionation can be modelled in either an open system or a closed system, which depends on whether there is an unlimited or limited supply of substrate (in this case DSi), respectively. In most open ocean regions silicon isotope fractionation can be accurately modelled using a closed system model, because summer stratification of the euphotic zone limits resupply of DSi to diatoms from depth. However, it is well documented[18, 19] that modern Antarctic waters are better represented by an open system model due to a vertical entrainment of DSi into surface waters during summer. A closed system better represents Subantarctic waters due to its stronger summer stratification[19].

Open system model equations:

$$\delta^{30}Si_{diat} = \delta^{30}Si(OH)_{4init} + \epsilon$$

$$\delta^{30}Si(OH)_4 = \delta^{30}Si(OH)_{4init} - \epsilon(1 - f)$$

Closed system model equations:

$$\delta^{30}Si_{diat} = \delta^{30}Si(OH)_{4init} - \epsilon\left(\frac{f \ln f}{1 - f}\right)$$

$$\delta^{30}Si(OH)_4 = \delta^{30}Si(OH)_{4init} + \epsilon \ln f$$

The Holocene and LGM outputs of their model are presented in Supplementary Figure 8. An open system model is used for the Antarctic and a closed system is shown for the Subantarctic. In the approach by Beucher et al. [17], a variable opal export was applied to each of the boxes by maintaining a constant silicon isotope composition of upwelled DSi (1.7 ‰) based on the assumption that this is unlikely to vary through time. However, this assumption may not have held with changes to the global silicon cycle through glacial-interglacial cycles. The modern deep ocean δ^{30}Si varies by approximately 0.2 ‰ between the Atlantic and Pacific and is relatively uniform within the Southern Ocean[22, 23]. However, an enhancement of Si trapping leading to an accumulation of DSi in the deep ocean during glacials (as described in the main text) may have increased the isotopic distillation that favours the accumulation of lighter isotopes in the deep ocean today[24]. Therefore, we allow the silicon isotopic composition of upwelled waters to vary, whilst assuming a given opal export from each box.

Another aspect of the model presented by Beucher et al.[17] is the input of an additional vertical DSi supply to the Subantarctic during the LGM reconstructions. This was deemed necessary to be able to account for the observed changes in opal flux between the LGM and Holocene. The justification for this additional vertical supply was the northward migration of the westerly winds during glacials, which may have driven more vigorous entrainment of DSi into Subantarctic surface waters.

The reconstructions presented here will focus on the hypothesised upwelling event during the Younger Dryas (YD). We will not present models reconstructing other intervals of the deglaciation (such as HS1) because several assumptions must be made about the environmental conditions and parameters to produce an accurate representation of the Si cycling and these assumptions are more likely to be valid during the YD. The assumptions include the opal export and isotope system model, the latter of which as discussed above may respond to the position of the westerlies and sea ice extent, which were likely more similar to modern conditions during the YD than earlier in the deglaciation.

Our second assumption is that the opal export from both the Antarctic and Subantarctic during the YD was the same as the Holocene (i.e. unchanged from that given in Fig. 4 of the main text). This assumption is more for simplicity and we acknowledge that on average opal flux records suggest the opal export may have been higher in the Southern Ocean during the YD compared to the Holocene[25]. As a result, the reconstructed DSi inputs and outputs may be interpreted as conservative.

The Antarctic and Subantarctic $\delta^{30}Si_{diat}$ used to fit these models were taken from the records presented in Fig. 3 in the main text. The records were analysed by linear interpolation resampling at 100-year intervals. The result of this resampling is presented in Supplementary Figure 9. The mean $\delta^{30}Si_{diat}$ within the core of the YD interval (11.8 – 12.5 ka) is 1.06 ‰ and 1.27 ‰ for the Antarctic and Subantarctic records, respectively. An example of one of the model outputs is given in Fig. 4 in the main text. Here we assume the YD had similar conditions to the modern, i.e. an open isotope system in the Antarctic, a closed system in the Subantarctic, and no additional vertical DSi supply to the Subantarctic.

We can allow the vertical input of DSi into the Subantarctic to vary reflecting increased vertical mixing in the Subantarctic demonstrated in the LGM reconstruction by Beucher et al.[17]. The silicon isotopic composition of this extra supply is forced to be identical to the vertical supply in the Antarctic as per Beucher et al.[17]. We can also allow each of the boxes (Antarctic and Subantarctic) to operate as open or closed isotope systems. The inputs (i.e. the vertical supply of DSi and its silicon isotope composition) to these solved models are presented in Supplementary Figure 10, and their corresponding outputs (i.e. the leakage of DSi to lower latitudes and its silicon isotope composition) are displayed in Supplementary Figure 11.

Of all the possible iterations of the Si dynamics in the Southern Ocean presented in Supplementary Figures 10 and 11, we suggest that the most likely are the conditions are those that produce an isotopic system similar to the modern Southern Ocean. These conditions, namely a southward position of the westerlies[29] and a removal of sea ice[30] and stratification[31] from the Antarctic, would likely favour an open system in the Antarctic and a closed system in the Subantarctic without any additional vertical supply of DSi to the Subantarctic. The mass balance model solution for this scenario has already been shown in Fig. 4 in the main text.

Based on these model outputs, we suggest that the concentration of DSi in the waters upwelled to the Antarctic mixed layer during the YD was 83 µM with an isotopic composition of 1.54 ‰. A large portion of this enhanced input of DSi to the Southern Ocean went unutilized leaving 24 µM of DSi to be transported from the Subantarctic to lower latitudes, an increase of 18 µM relative to modern. However, as mentioned above, this is likely an underestimate as we have assumed the same opal export for the YD as the modern. An increase in opal export would require a greater supply of DSi and consequently a greater leakage to lower latitudes. Overall this exercise corroborates our main interpretation of the $\delta^{30}Si_{diat}$ data given in the main text, that the YD was a period of greater DSi flux to the Southern Ocean and enhanced leakage of DSi to lower latitudes.

Supplementary Note 6: Global deglacial burial of silica

Using silicon isotope records from diatoms and sponges, we lay out a hypothesis in the main text suggesting that during the last glacial maximum the whole ocean Si inventory was greater than today thanks to lower Si burial induced by an Fe-regulated decline in Si uptake by diatoms and a reduction in diatom productivity in the ice-covered Antarctic. To investigate whether opal burial increased across the deglaciation we have compiled ^{230}Th-normalised opal flux records (Supplementary Table 4). This list of records is not exhaustive but spans the world's major open ocean opal deposition regions[38] and includes only records with at least three data points across the deglaciation (10 – 18 ka). The records were analysed by linear interpolation resampling at 100-year intervals. The mean opal fluxes across the Holocene (H, 0 – 10 ka), deglaciation (D, 10 – 18 ka) and late deglaciation (LD, 10 – 15 ka) are displayed in Supplementary Table 4, along with the differences between these means (D – H, LD – H) and the percentage change with respect to the Holocene.

Based on this simple analysis, most records (30/40) show a greater opal flux across the whole deglaciation (10 -18 ka) and even more (33/40) across the late deglaciation (10 – 15 ka) than the Holocene. On average the records display a 35 % greater opal flux during the whole deglaciation and a 49 % greater opal flux during the late deglaciation with respect to the Holocene. This global change is comparable to the average of the Southern Ocean records along (34 % and 51 % for the whole and late deglaciation periods, respectively). This is understandable as the largest recorded opal fluxes and deglacial opal flux changes are within Southern Ocean records.

By taking these first order estimates of changes in global opal burial we can estimate the change in the global oceanic Si inventory across the deglaciation. Assuming no change in terrestrial DSi input, a 35%

increase in the modern open ocean diatom burial (modern = 3.04+/-1.54 Tmol/yr [39]) would equal an opal burial rate of 4.104 Tmol/yr. The increased burial would result in the additional removal of 8512 Tmol Si from the ocean across the 8000-year deglacial interval (10 – 18 ka). With the modern ocean DSi inventory estimated to be 97000 Tmol DSi[39], the removal of 8512 Tmol Si across the deglaciation suggests the glacial Si inventory was 105512 Tmol Si, or ~9 % greater than the modern. This estimate is remarkably comparable to the 12 % and 6 – 19 % values given by Jochum et al.[40] and Ellwood et al.[41], respectively for their estimates of the reduction in Si inventory across the deglaciation. It should be noted that ours is likely an underestimate given that the DSi input to the ocean has been suggested to have decreased[42].

Location[referemce]	Core	Mean Holocene (H) (0 - 10 ka)	Mean deglacial (D) (10 - 18 ka)	Mean Late deglacial (LD) (10 - 15 ka)	D - H	Location mean D - H	Location mean D as % of H	LD - H	Location mean LD - H	Location mean LD as % of H
Equatorial Atlantic[32]	RC24-01	0.01	0.09	0.07	0.07	0.04	224 %	0.06	0.03	207 %
	RC24-07	0.06	0.12	0.11	0.06			0.05		
	RC24-12	0.04	0.06	0.06	0.02			0.02		
	RC16-66	0.03	0.05	0.05	0.02			0.02		
	RC13-189	0.01	0.04	0.03	0.03			0.02		
	V30-40	0.03	0.06	0.05	0.03			0.03		
	V22-182	0.03	0.07	0.06	0.04			0.03		
Equatorial Pacific[33]	V19-30	0.26	0.30	0.35	0.04	0.10	135 %	0.09	0.10	137 %
	V21-40	0.09	0.06	0.07	-0.04			-0.03		
	RC13-140	0.07	0.06	0.06	0.00			0.00		
	RC11-238	0.12	0.12	0.14	0.00			0.02		
	RC13-114	0.13	0.12	0.13	-0.02			0.00		
	TT013-PC72	0.06	0.06	0.07	0.00			0.01		
	RC17-177	0.02	0.02	0.02	0.00			0.00		
	MD2138	0.04	0.06	0.05	0.02			0.01		
	MW91-9	0.02	0.03	0.03	0.00			0.00		
Equatorial Pacific[34]	ODP 1240	1.98	2.95	2.91	0.96			0.93		
North Pacific[35]	ODP 887B	0.26	0.11	0.14	-0.15	0.07	126 %	-0.12	0.13	150 %
	JPC17	0.21	0.30	0.32	0.10			0.11		
	RC10-196	0.27	0.46	0.58	0.19			0.31		
	PC13	0.35	0.47	0.62	0.12			0.27		
	ODP 882	0.19	0.27	0.25	0.08			0.07		
Southern Ocean[26]	PS1768-8	0.98	2.19	2.80	1.21	0.25	134 %	1.82	0.37	151 %
Southern Ocean[25]	TN057 13PC4	1.84	3.36	4.15	1.52			2.31		
Southern Ocean[36]	E27-23	0.57	0.65	0.81	0.08			0.24		
Southern Ocean[20]	MD94102	0.06	0.06	0.06	0.01			0.00		
	MD94104	0.23	0.22	0.20	-0.01			-0.03		
	MD88-769	0.06	0.11	0.08	0.05			0.02		
	MD88-770	0.08	0.26	0.16	0.18			0.08		
	MD84-552	1.58	0.82	1.10	-0.76			-0.48		
Southern Ocean[21]	NBP9802-6PC1	1.60	2.22	2.04	0.63			0.44		
	NBP9802-5GC1	0.99	0.84	0.94	-0.15			-0.04		
	E17-9	0.63	0.56	0.63	-0.08			0.00		
Southern Ocean[10, this study]	MD88-773	0.63	0.61	0.76	-0.02			0.13		
Southern Ocean[37]	PS1768	0.87	2.28	2.71	1.41			1.84		
	PS1772	0.28	0.12	0.11	-0.16			-0.17		
	PS1754	0.12	0.21	0.16	0.09			0.04		
	PS1756	0.42	1.58	1.22	1.16			0.8		
	PS2082	0.09	0.22	0.15	0.12			0.06		
Southern Ocean[this study]	MD84-551	2	1.14	1.55	-0.86			-0.45		
					Total	0.15	135 %		0.21	149 %

Supplementary Table 4. Estimates of changes in global burial of opal across the deglaciation. All units are g cm^{-2} kyr^{-1} unless specified otherwise.

Supplementary References

1. Key, R.M. et al. A global ocean carbon climatology: Results from Global Data Analysis Project (GLODAP). *Global Biogeochem. Cycles*. **18**, 1–23. (2004).

2. Stuiver, M. & Reimer, P.J. Extended ^{14}C Data Base and Revised CALIB 3.0 ^{14}C Age Calibration Program. Radiocarbon. **35**, 215–230. (1993).

3. Labracherie, M. et al. The last deglaciation in the Southern Ocean. *Paleoceanography.* **4**, 629–638. (1989).

4. Pichon, J.-J. et al. Surface water temperature changes in the high latitudes of the Southern Hemisphere. *Paleoceanography*. **7**, 289–318. (1992).

5. Gottschalk, J. et al. Radiocarbon measurements of small-szie foraminiferal samples with the mini carbon dating system (MICADAS) at the University of Bern: Implications for paleoclimate reconstructions. *Radiocarbon.* **60**, 469–491. (2018).

6. Mazaud, A. et al. Variations of the ACC-CDW during MIS3 traced by magnetic grain deposition in midlatitude South Indian Ocean cores: Connections with the northern hemisphere and with central Antarctica. *Geochemistry, Geophys. Geosystems*. **8**(5). (2007).

7. Mazaud, A. & Michel, E. Les Rapports des Campagnes à la Mer: MD189/INDIEN SUD-2 à bord du R/V Marion Dufresne. (2012).

8. Pugh, R.S. et al. Circum-Antarctic age modelling of Quaternary marine cores under the Antarctic Circumpolar Current: Ice-core dust-magnetic correlation. *Earth Planet. Sci. Lett*. **284**, 113–123. (2009).

9. Bareille, G. et al. Origin of detrital fluxes in the southeast Indian Ocean during the last climatic cycles. *Paleoceanography.* **9**, 799–819. (1994).

10. Francois, R. et al. Contribution of Southern Ocean surface-water stratification to low atmospheric CO_2 concentrations during the last glacial period. *Nature*. **389**, 929–935. (1997).

11. Lambert, F. et al. Centennial mineral dust variability in high-resolution ice core data from Dome C, Antarctica. *Clim. Past.* **8**, 609–623. (2012).

12. Chase, Z., Kohfeld, K. E., & Matsumoto, K. Controls on biogenic silica burial in the Southern Ocean. *Global Biogeochemical Cycles*, **29**, 1599–1616. (2015).

13. Fripiat, F. et al. Diatom-induced silicon isotopic fractionation in Antarctic sea ice. *Journal of Geophysical Research: Biogeosciences*, **112**(2). (2007).

14. Crosta, X., Pichon, J. & Burckle, L.H.Application of modern analog technique to marine Antarctic diatoms: Reconstruction of maximum sea-ice extent at the Last Glacial Maximum. *Paleoceanography*. **13**, 284–297. (1998).

15. Sutton, J.N. et al. Species-dependent silicon isotope fractionation by marine diatoms. *Geochim. Cosmochim. Acta.* **104**, 300–309. (2013).

16. Horn, M. G. et al. Southern Ocean nitrogen and silicon dynamics during the last deglaciation. *Earth Planet. Sci. Lett*, **310**(3–4), 334–339. (2011).

17. Beucher, C. P., Brzezinski, M. A., & Crosta, X. Silicic acid dynamics in the glacial sub-Antarctic: Implications for the silicic acid leakage hypothesis. *Global Biogeochemical Cycles*, **21**(3), 1–13. (2007).

18. Varela, D. E., Pride, C. J., & Brzezinski, M. A. Biological fractionation of silicon isotopes in Southern Ocean surface waters. *Global Biogeochemical Cycles*, **18**, 1–8. (2004).

19. Fripiat, F. et al. Isotopic constraints on the Si-biogeochemical cycle of the Antarctic Zone in the Kerguelen area (KEOPS). *Marine Chemistry*, **123**(1–4), 11–22. (2011).

20. Dezileau, L., Reyss, J. L., & Lemoine, F. Late Quaternary changes in biogenic opal fluxes in the Southern Indian Ocean. *Marine Geology*, **202**(3–4), 143–158. (2003).

21. Chase, Z. et al. Accumulation of biogenic and lithogenic material in the Pacific sector of the Southern Ocean during the past 40,000 years. *Deep Sea Res. II*, **50**, 799–832. (2003).

22. De Souza, G. F. et al. Silicon stable isotope distribution traces Southern Ocean export of Si to the eastern South Pacific thermocline. *Biogeosciences*, **9**(11), 4199–4213. (2012).

23. De Souza, G. F. et al. Deconvolving the controls on the deep ocean's silicon stable isotope distribution. *Earth Planet. Sci. Lett*. **398**, 66–76. (2014).

24. Brzezinski, M. A., & Jones, J. L. Coupling of the distribution of silicon isotopes to the meridional overturning circulation of the North Atlantic Ocean. *Deep Sea Res. II*, **116**, 79–88. (2015).

25. Anderson, R. F. et al. Wind-driven upwelling in the Southern Ocean and the deglacial rise in atmospheric CO_2. *Science*, ***323***, 1443–1448. (2009).

26. Robinson, R.S. et al. The changing roles of iron and vertical mixing in regulating nitrogen and silicon cycling in the Southern Ocean over the last glacial cycle. *Paleoceanography.* **29**(12), 1179–1195. (2014).

27. Rousseau, J. et al. Estimates of late Quaternary mode and intermediate water silicic acid concentration in the Pacific Southern Ocean. *Earth Planet. Sci. Lett*. **439**, 101–108. (2016).

28. Abelmann, A. et al. The seasonal sea-ice zone in the glacial Southern Ocean as a carbon sink. *Nature Commun.* **6**, 8136. (2015).

29. Kohfeld, K. E. et al. Southern Hemisphere westerly wind changes during the Last Glacial Maximum: paleo-data synthesis. *Quaternary Science Reviews*, **68**, 76–95. (2013).

30. Xiao, W., Esper, O. & Gersonde, R. Last Glacial - Holocene climate variability in the Atlantic sector of the Southern Ocean. *Quat. Sci. Rev*. **135**, 115–137. (2016).

31. Studer, A. S. et al. Antarctic Zone nutrient conditions during the last two glacial cycles. *Paleoceanography*. **30**(7). (2015).

32. Bradtmiller, L.I. et al. Opal burial in the equatorial Atlantic Ocean over the last 30 ka: Implications for glacial-interglacial changes in the ocean silicon cycle. *Paleoceanography*. **22**. (2007).

33. Bradtmiller, L. I. et al. Diatom productivity in the equatorial Pacific Ocean from the last glacial period to the present: A test of the silicic acid leakage hypothesis. *Paleoceanography*, **21**(4). (2006).

34. Pichevin, L. E. et al. Enhanced carbon pump inferred from relaxation of nutrient limitation in the glacial ocean. *Nature*, **459**, 1114–1117. (2009).

35. Kohfeld, K. E., & Chase, Z. (2011). Controls on deglacial changes in biogenic fluxes in the North Pacific Ocean. *Quaternary Science Reviews*, **30**, 3350–3363. (2011).

36. Bradtmiller, L. I. et al. Comparing glacial and Holocene opal fluxes in the Pacific sector of the Southern Ocean. *Paleoceanography*, **24**(2). (2009).

37. Frank, M. et al. Similar glacial and interglacial export bioproductivity in the Atlantic sector of the Southern Ocean: Multiproxy evidence and implications for glacial atmospheric CO_2. *Paleoceanography*, **15**(6), 642–658. (2000).

38. DeMaster, D. J. The accumulation and cycling of biogenic silica in the Southern Ocean: revisiting the marine silica budget. *Deep Sea Res. II.* **49**(16), 3155–3167. (2002).

39. Tréguer, P.J. & De La Rocha, C.L., 2013. The world ocean silica cycle. *Ann. Rev. Mar. Sci*. **5**, 477–501. (2013).

40. Jochum, K. P. et al. Whole-Ocean Changes in Silica and Ge/Si Ratios During the Last Deglacial Deduced From Long-Lived Giant Glass Sponges. *Geophysical Research Letters*. **4**, 11555–11564. (2017).

41. Ellwood, M.J., Wille, M. & Maher, W. Glacial silicic acid concentrations in the Southern Ocean. *Science.* **330**, 1088–1091. (2010).

42. Frings, P. J. et al. The continental Si cycle and its impact on the ocean Si isotope budget. *Chemical Geology*, **425**, 12–36. (2016).

Supplementary Figures

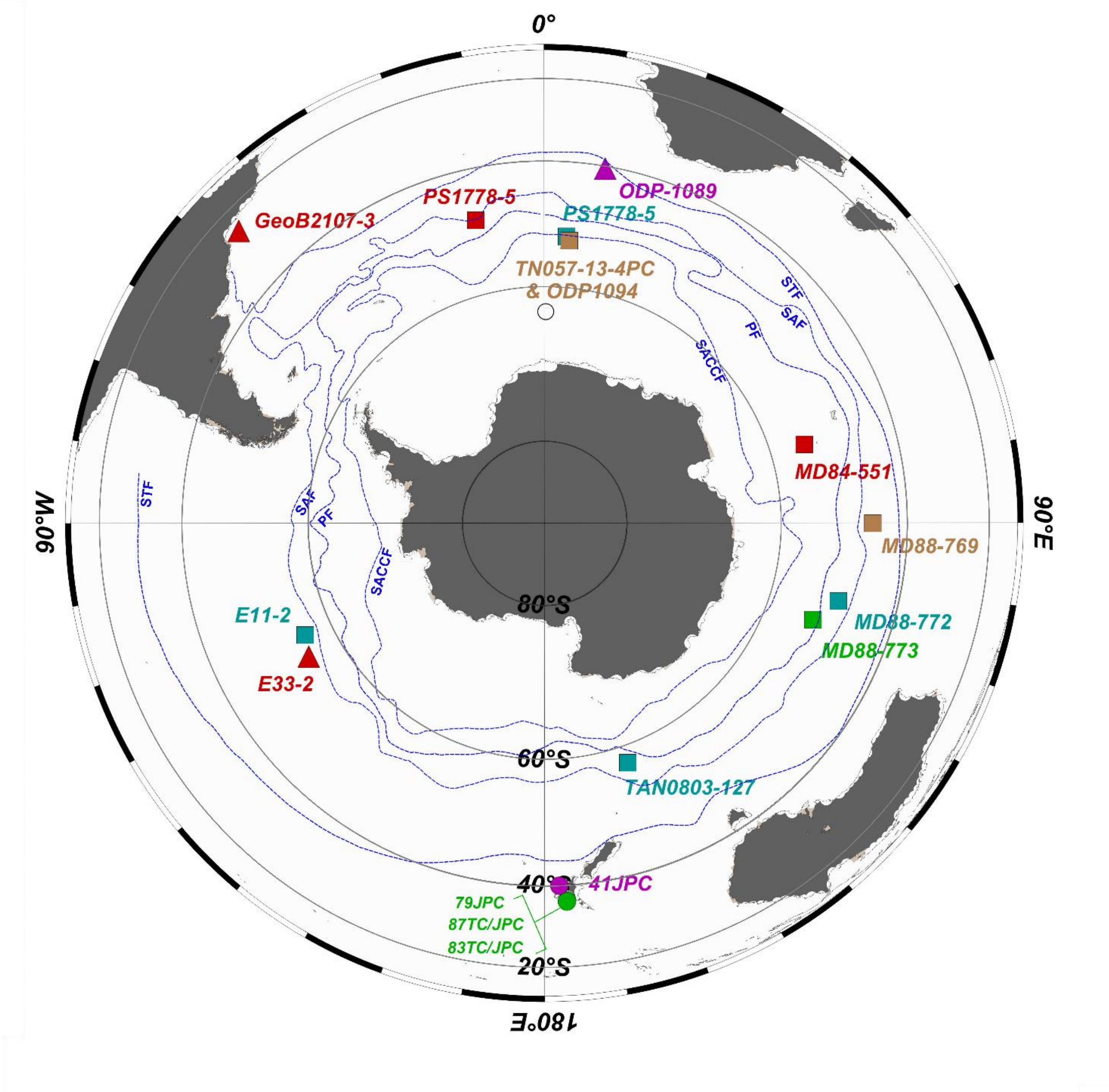

Supplementary Figure 1. Map of core locations for records used in Fig. 3 (main text). Squares denote locations from which $\delta^{30}Si_{diat}$ data was taken (Fig. 3A, main text), triangles are those from which $\delta^{30}Si_{sponge}$ data was taken (Figs. 3C & D, main text) and circles are those from which $\delta^{13}C_{Cib}$ data was taken (Fig. 3E, main text).

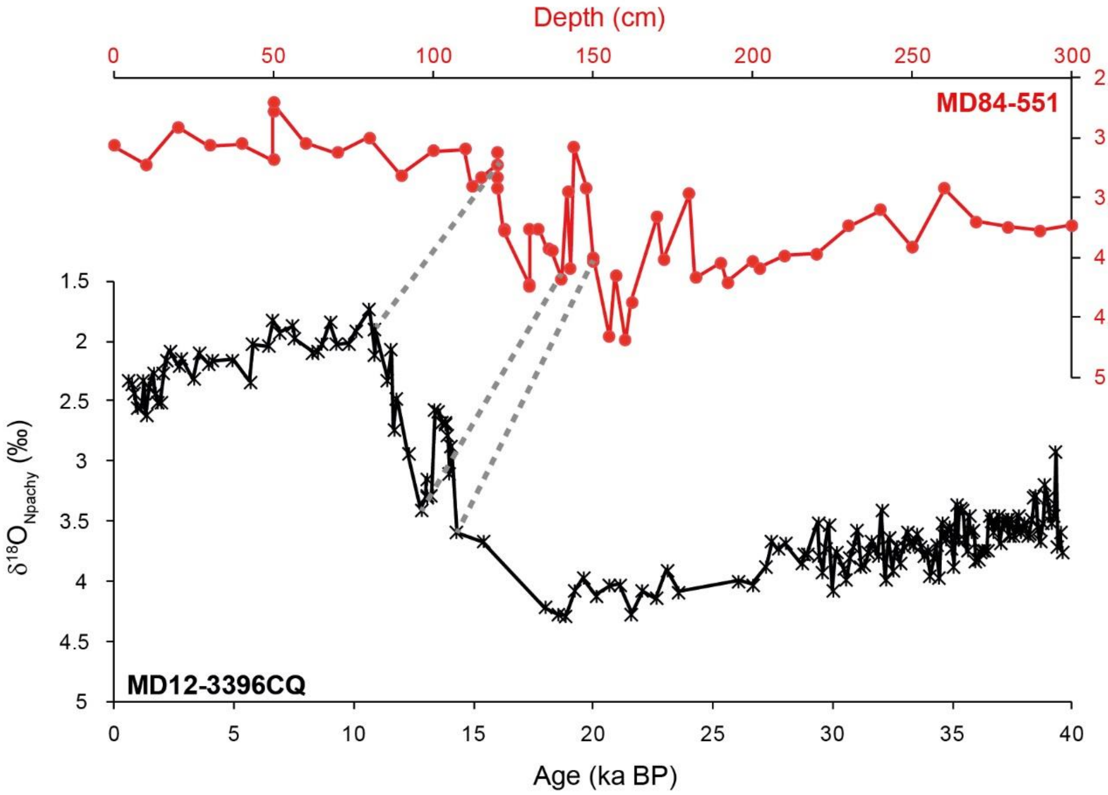

Supplementary Figure 2. Stratigraphic alignment for MD84-551. Age control points aligning the planktic foraminiferal (*Neogloboquadrina pachyderma*) $\delta^{18}O$ records of MD84-551[3,4] and MD12-3396CQ[5].

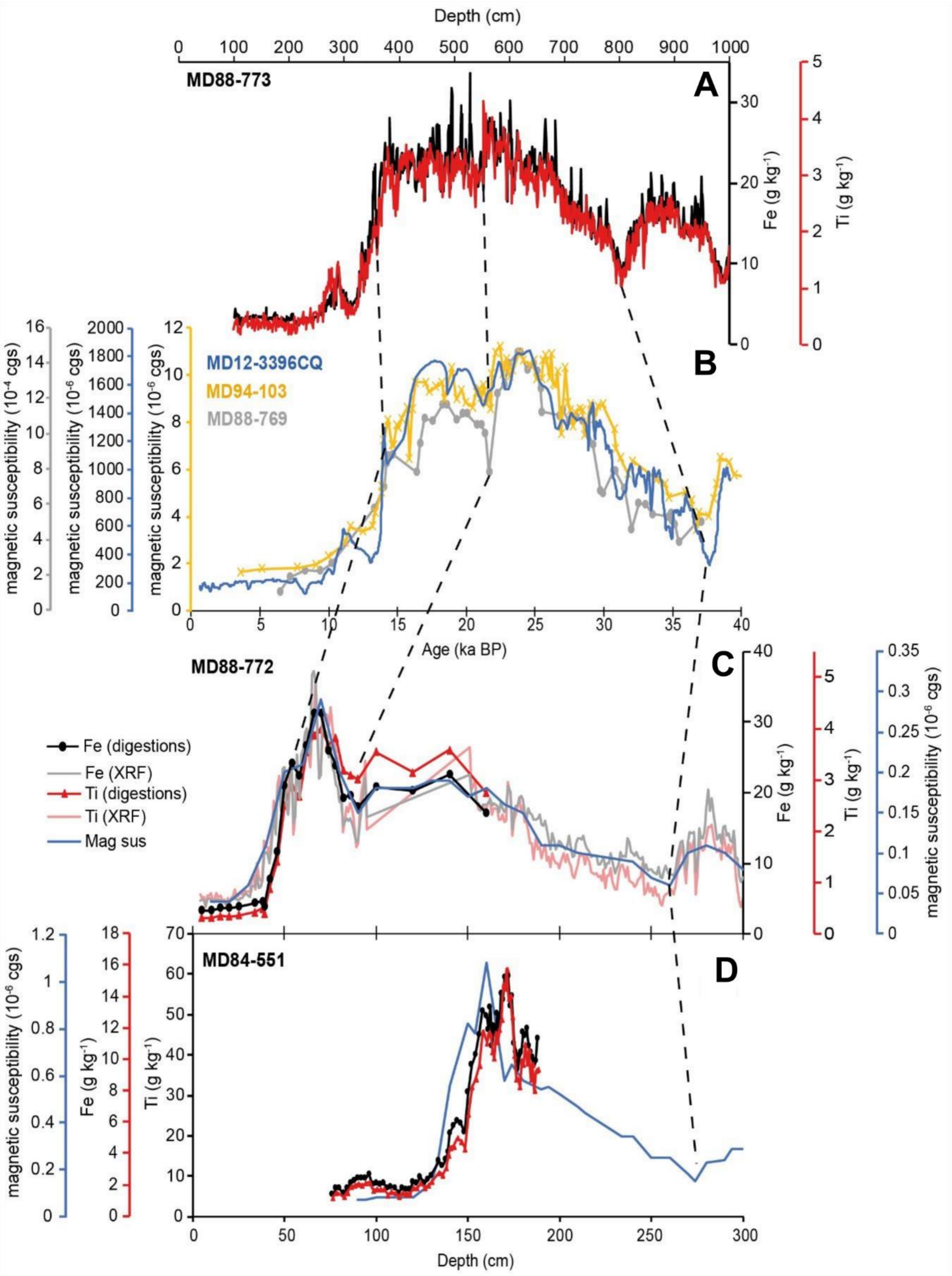

Supplementary Figure 3. Stratigraphic alignment for MD84-551, MD88-773 and MD88-772. Age control points aligning the magnetic susceptibility and lithogenic detritus proxies from MD88-773 **(A),** MD88-772 **(B)** and MD84-551 **(D)** with nearby, independently dated magnetic susceptibility records from MD94-103[6], MD88-769[6] and MD12-3396CQ[7] **(C)**. Black dashed lines indicate age control points.

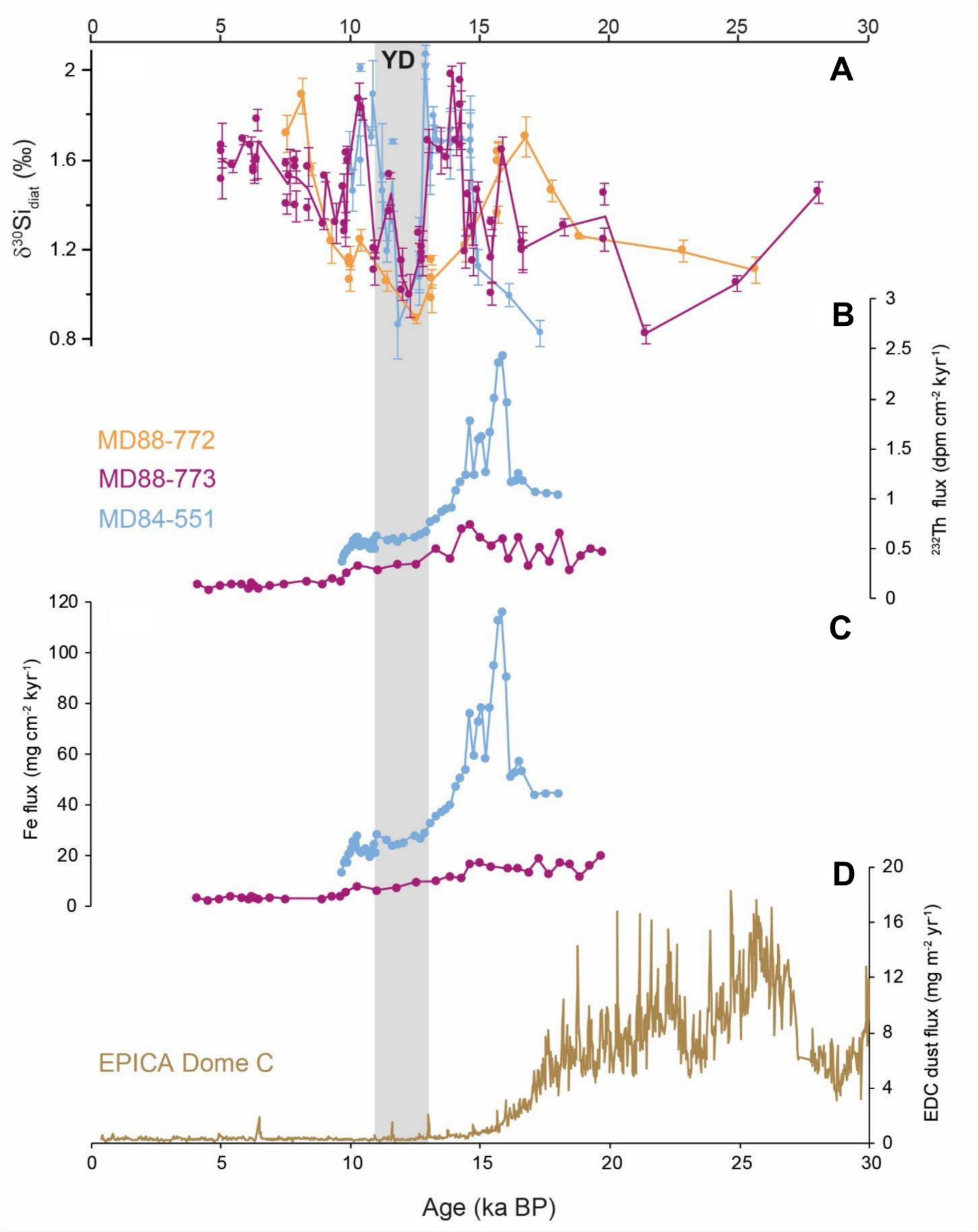

Supplementary Figure 4. Lithogenic inputs across the deglaciation compared to Antarctic dust fluxes and silicon isotope records. (A) Diatom silicon isotope ($\delta^{30}Si_{diat}$) records from MD84-551, MD88-773 and MD88-772. Error bars are ±1SE **(B)** ^{230}Th-normalised ^{232}Th flux data from MD88-773[10] and MD84-551 as a proxy for lithogenic material input. **(C)** ^{230}Th-normalised Fe flux data from MD88-773[10] and MD84-551 as a proxy for lithogenic material input. **(D)** Antarctic dust flux record from EPICA Dome C[11].

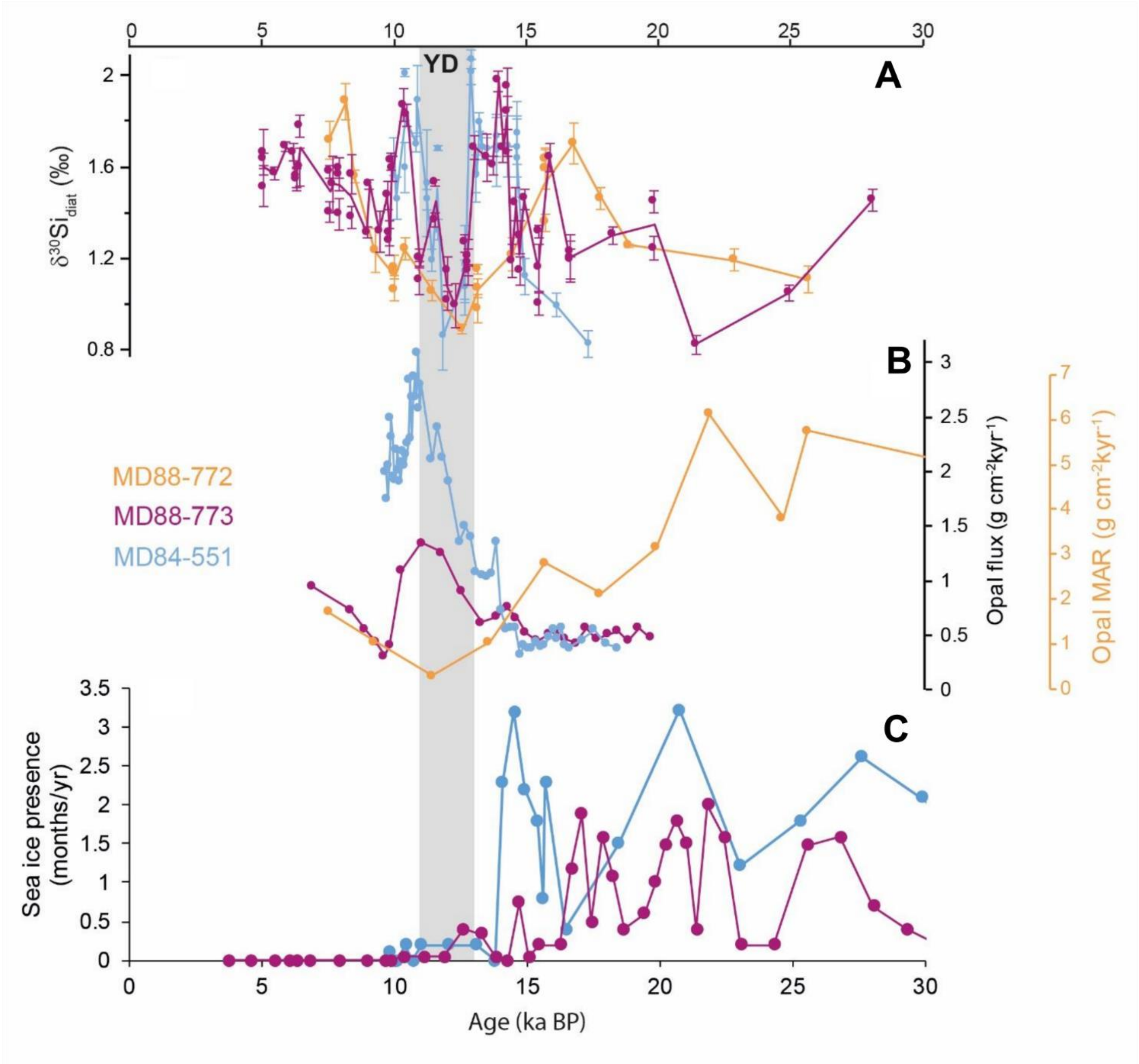

Supplementary Figure 5. Demonstration of the relationship between sea ice decline and silicon cycle changes. (A) Diatom silicon isotope ($\delta^{30}Si_{diat}$) records from MD84-551, MD88-773 and MD88-772. Error bars are ±1SE. **(B)** ^{230}Th-normalised opal flux records from MD84-551 and MD88-773 (^{230}Th-normalisation data from Francois et al (1997) and opal mass accumulation rate (MAR) from MD88-772. **(C)** Sea ice presence estimated using a modern [illegible]

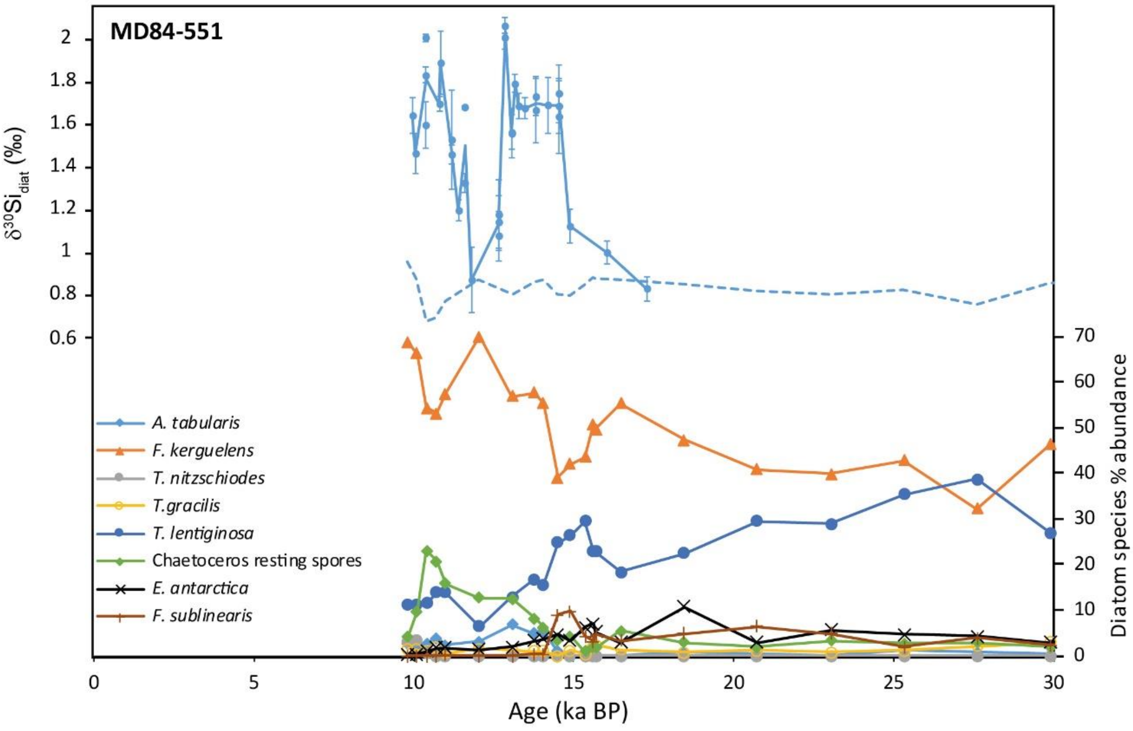

Supplementary Figure 6. Diatom silicon isotope ($\delta^{30}Si_{diat}$) and diatom assemblage records from MD84-551. The dashed blue line is the reconstructed δ^{30}Si variability based on species effects ($\delta^{30}Si_{model}$). Error bars are ±1SE.

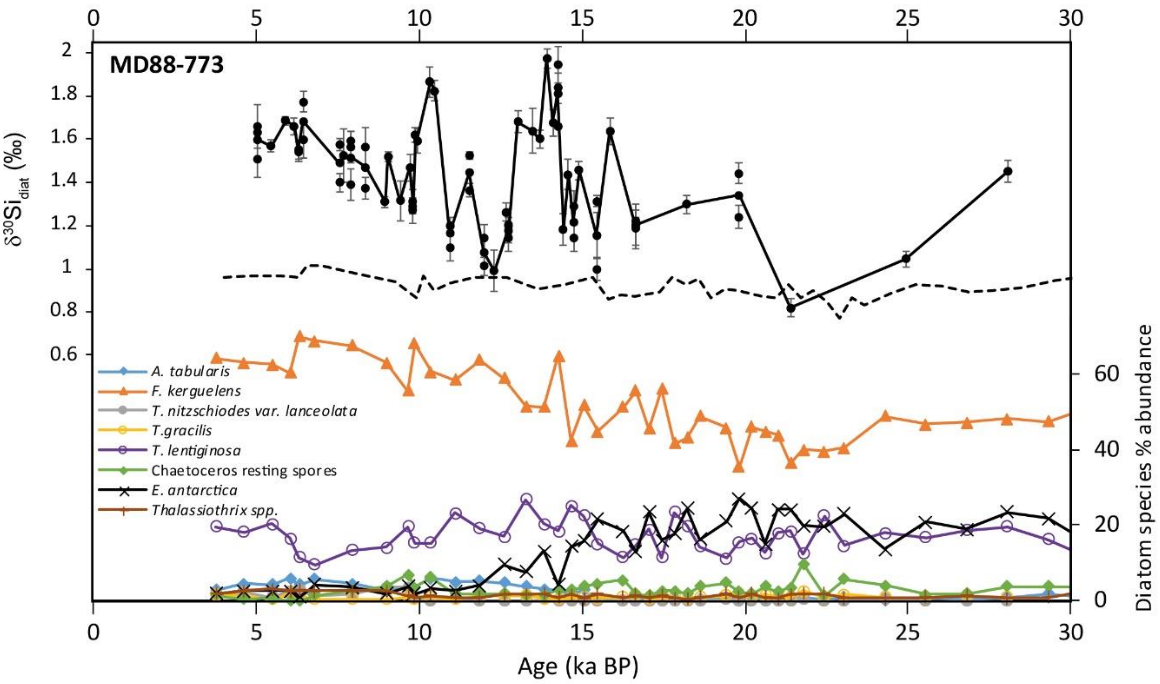

Supplementary Figure 7. Diatom silicon isotope ($\delta^{30}Si_{diat}$) and diatom assemblage records from MD88-773. The dashed black line is the reconstructed δ^{30}Si variability based on species effects ($\delta^{30}Si_{model}$). Error bars are ±1SE.

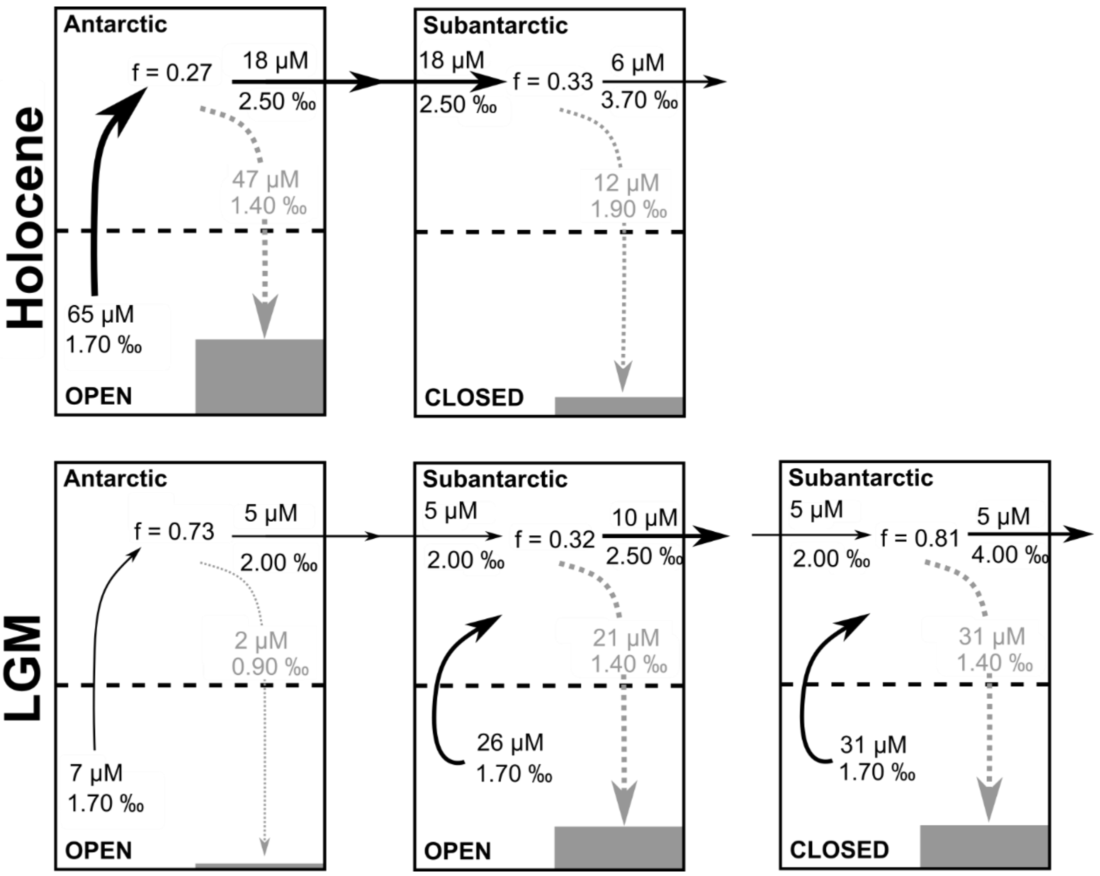

Supplementary Figure 8. Solution to mass balance model of Si cycling in the Southern Ocean at the Holocene and LGM from Beucher et al (2007). The LGM solutions require an additional input of DSi into the Subantarctic in order to account for the observed increase in opal fluxes within the Subantarctic at the LGM[20, 21].

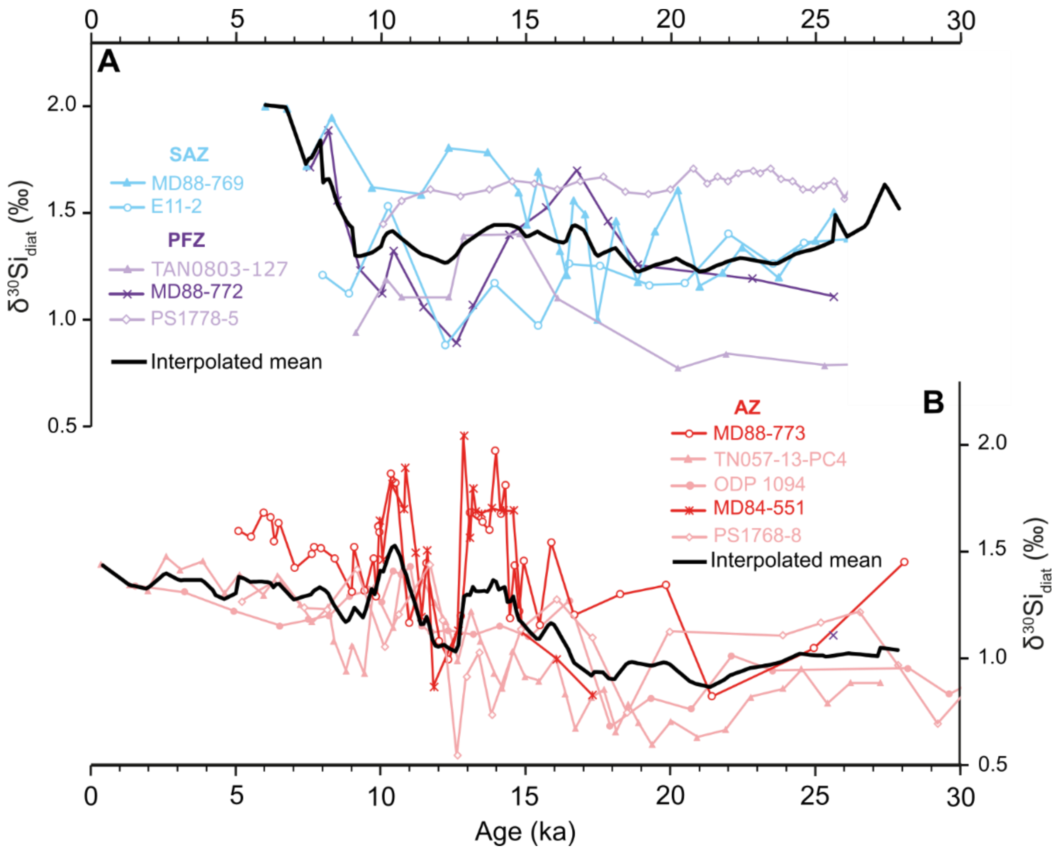

Supplementary Figure 9. A plot showing the interpolated means for the Antarctic and Subantarctic records shown in Fig. 3 (main text). Diatom silicon isotope ($\delta^{30}Si_{diat}$) records from the Subantarctic **(A)** and Antarctic **(B)**. MD88-769[17]. E11-2 & ODP1094[26]. TAN0803-127[27]. PS1778-5 & PS1768-8[28]. TN057-13-PC4[16]. Black lines in each plot are the means from each zone (Antarctic or Subantarctic) determined by resampling linearly interpolated records at 100 year intervals.

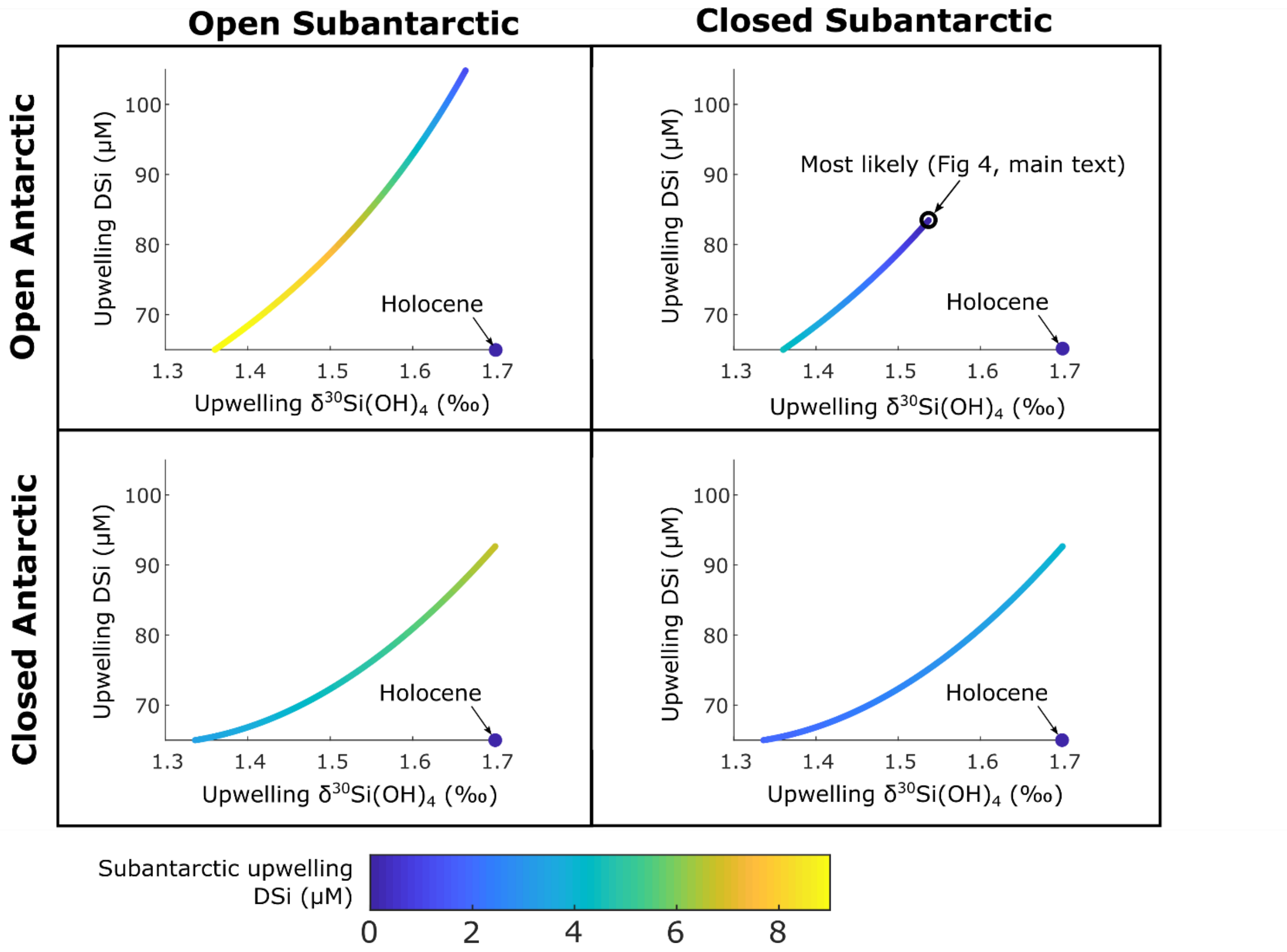

Supplementary Figure 10. The DSi concentration and isotopic composition of Antarctic upwelling during the YD. These are estimates from the solutions to all the mass balance models of Si cycling in the Southern Ocean and include all combinations of open and closed isotope models (individual panels). The colouring of each plot denotes the additional DSi input into the Subantarctic. The isotopic composition of this additional input is identical to the input into the Antarctic. The scenario which was suggested to be most compatible with conditions during the YD is highlighted ("Most likely") and is depicted fully in Fig. 4 within the main text.

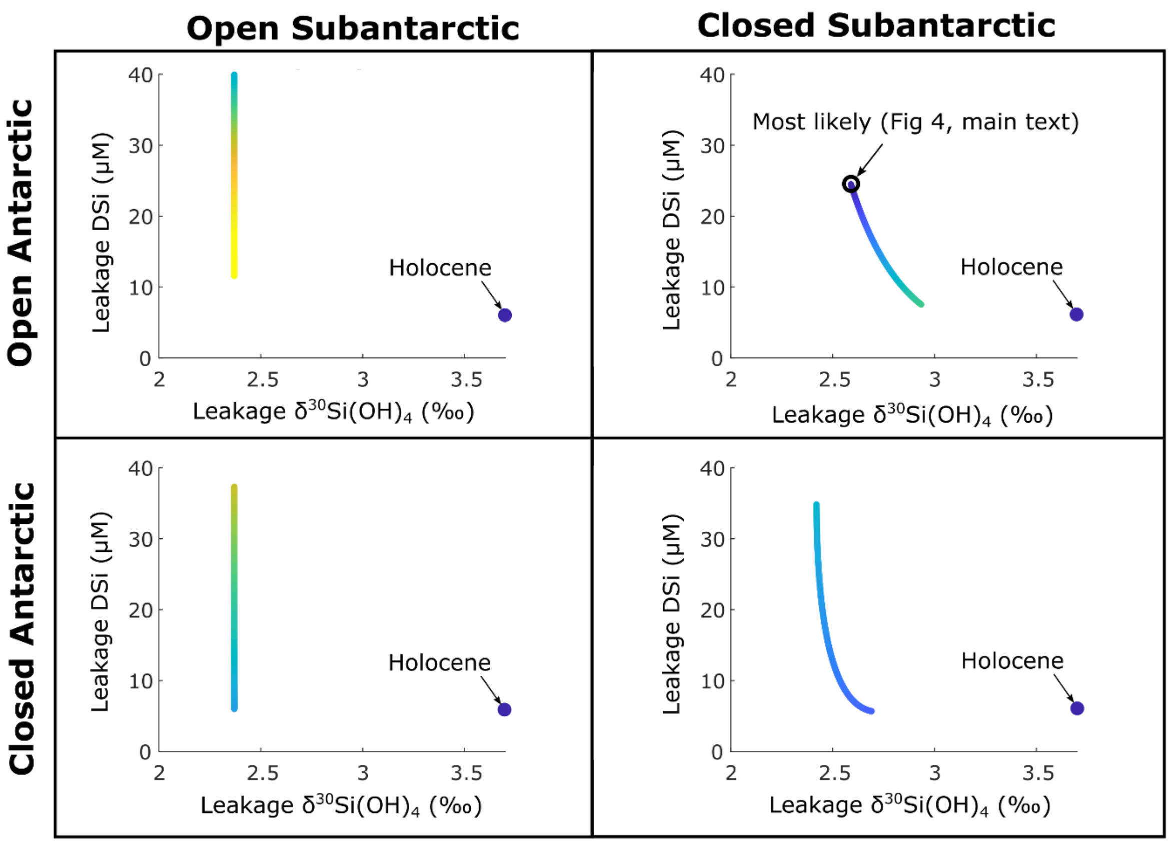

Supplementary Figure 11. The DSi concentration and isotopic composition of waters exiting the Subantarctic to low latitudes (leakage) during the YD. These are estimates from the solutions to all the mass balance models of Si cycling in the Southern Ocean and includes all combinations of open and closed isotope models (individual plots). The colouring of each plot denotes the additional DSi input into the Subantarctic. The isotopic composition of this additional input is identical to the input into the Antarctic. The scenario which was suggested to be most compatible with conditions during the YD is highlighted ("Most likely") and is depicted fully in Fig. 4 within the main text.
